# Autoimmune Bullous Dermatosis Following COVID-19 Vaccination: A Series of Five Cases

**DOI:** 10.7759/cureus.23127

**Published:** 2022-03-13

**Authors:** Fouzia Hali, Lamiae Araqi, Farida Marnissi, Ahlam Meftah, Soumiya Chiheb

**Affiliations:** 1 Dermatology, Ibn Rochd University Hospital Center, Casablanca, MAR; 2 Anatomical Pathology, Ibn Rochd University Hospital Center, Casablanca, MAR; 3 Pharmacology, Ibn Rochd University Hospital Center, Casablanca, MAR

**Keywords:** imputability, immunology, covid-19 vaccine, superficial pemphigus, pemhigus vulgaris, bullous pemphigoid, autoimmune bullous dermatosis

## Abstract

Autoimmune bullous diseases (AIBDs) are a heterogeneous group of diseases characterized by cutaneous and mucosal vesicles, blisters, and erosions. Several factors can trigger this disease, including vaccines; but this entity remains very rare. We hypothesized that vaccination against coronavirus disease 2019 (COVID-19) could trigger an immunological response in genetically predisposed individuals. We report five cases of new-onset autoimmune bullous diseases triggered by the COVID-19 vaccine. Clinical and histopathological examinations confirmed the diagnosis of bullous pemphigoid (BP) in three patients and pemphigus in the other two. According to the French method of imputability, the pharmacovigilance investigation showed an I5B4 causality assessment score for the vaccines, interpreted as highly probable, for all the patients. The diagnosis of vaccine-induced autoimmune bullous dermatosis was highly suspected. One patient's condition improved by dermocorticoids alone, while the other four required oral corticosteroid therapy at 0.5 mg/kg/day, which led to a favorable outcome.

## Introduction

Autoimmune bullous diseases (AIBDs) are a heterogeneous group of diseases that often manifest as cutaneous and mucosal vesicles, blisters, and erosions. The pathogenic mechanism behind the condition involves the presence of autoantibodies targeting desmosomes and hemidesmosomes. These structural proteins are targeted and are intraepidermal in pemphigus, causing the blisters to be superficial and fragile, while they are subepidermal in bullous pemphigoid (BP), leading to the formation of tense bullae. Several factors are known to trigger AIBD, including vaccines [1]. The World Health Organization declared Coronavirus disease 2019 (COVID-19) as a global pandemic in March 2020, and millions of people have been affected by the disease worldwide. A vaccination campaign was launched in Morocco in January 2021, resulting in a notable increase in autoimmune diseases [2]. In this report, we discuss five cases of new-onset AIBD triggered by the COVID-19 vaccine, three of which had BP while the other two had pemphigus. In all five cases, the diagnosis was confirmed by pharmacovigilance investigations.

## Case presentation

Case 1

**Figure 1:**
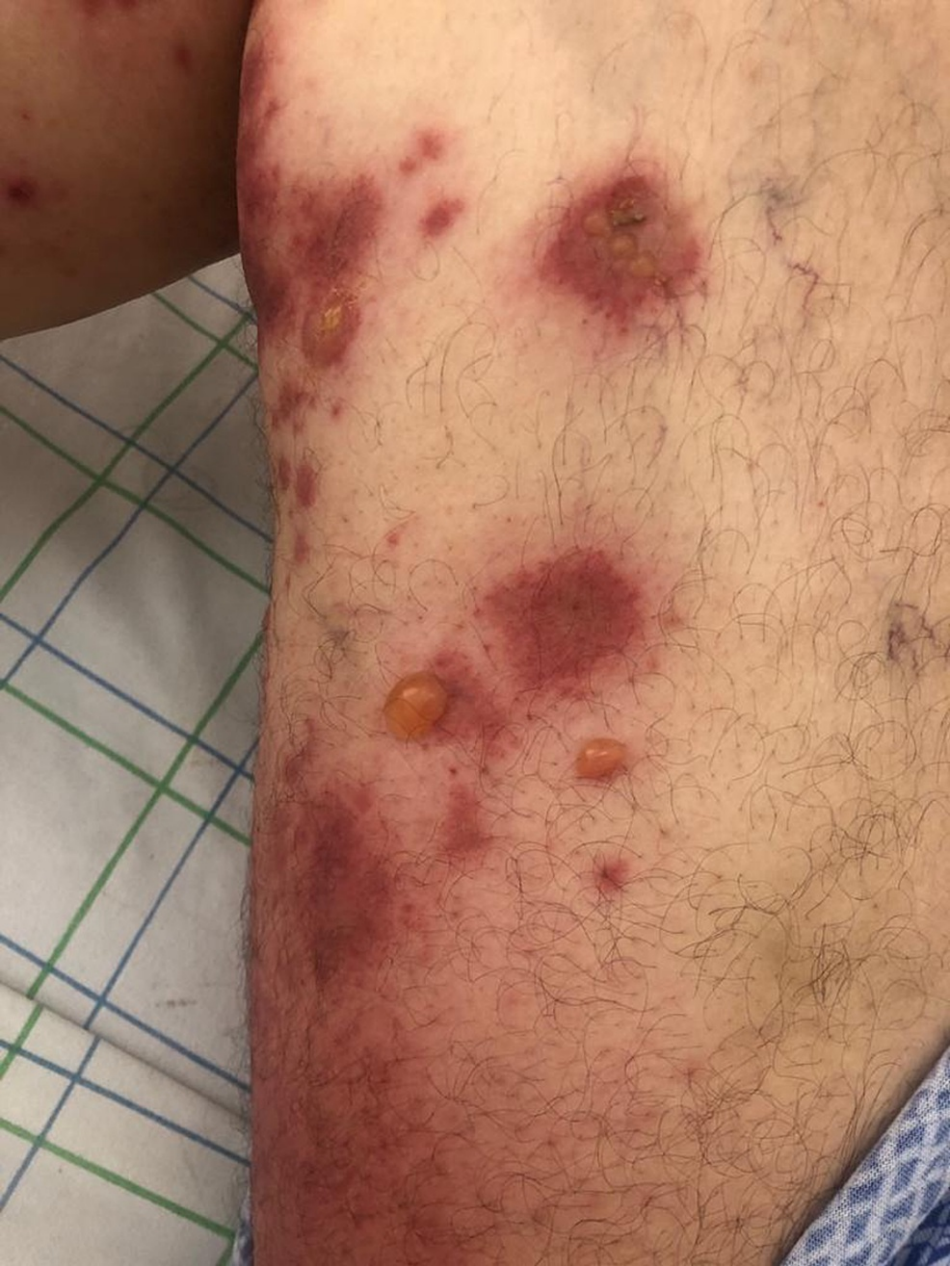
Tense bullae with clear content on urticarial skin on the right thigh

**Figure 2:**
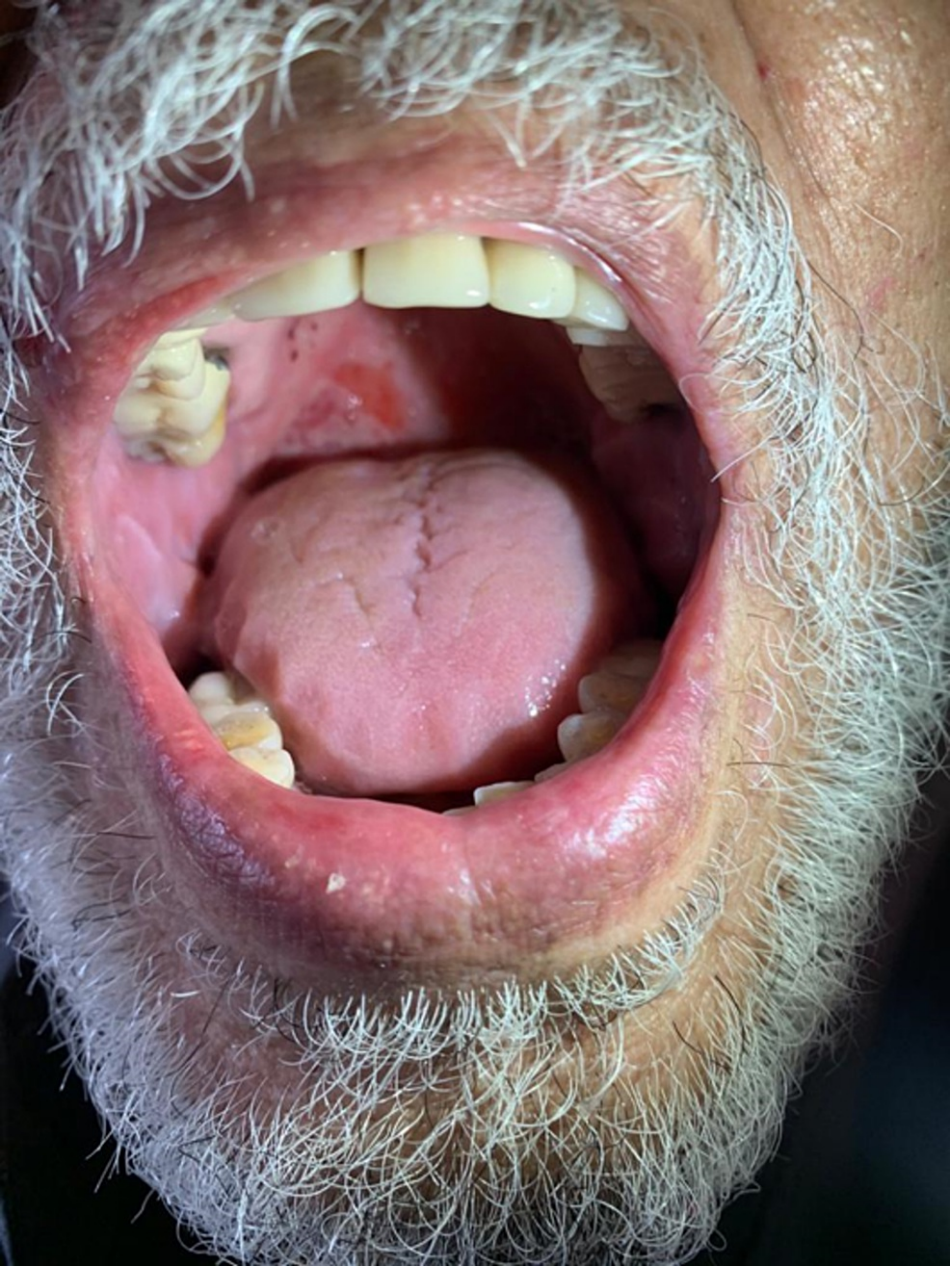
Erosions on the palate

A 51-year-old man was admitted for the management of extensive bullous eruption, which had appeared seven days after the administration of the Oxford AstraZeneca COVID-19 vaccine’s first booster. He had no specific medical history and was not taking any other medications. The clinical examination showed the presence of diffuse tense bullae associated with urticarial plaques on the trunk, back, and legs; post-bullous erosions and cocarde-like lesions affecting 70% of the skin surface were observed. The Nikolsky sign was positive (Figure 1). The patient also had multiple erosions on the buccal mucosa (Figure 2).

Histological examination showed subepidermal split with a superficial perivascular inflammatory infiltrate and predominant eosinophils. Direct immunofluorescence detected tissue-bound autoantibodies with a linear C3 pattern at the basement membrane zone. The indirect immunofluorescence performed on salt-split normal human skin substrate with the patient's serums detected circulating immunoglobulin G (IgG) (BP180 antibodies), consistent with BP. The remaining laboratory analyses yielded normal results, except for a high count of eosinophils. We notified the pharmacovigilance department and an immediate thorough investigation was started, which showed an I5B4 causality assessment score for the vaccine according to the French method of imputability [3]. The diagnosis of vaccine-induced BP was highly suspected. The patient was put on prednisone 30 mg daily (0.5 mg/kg daily) for six weeks, subsequently reduced by 5 mg every week, and then fully stopped. A complete resolution of the blisters was achieved after only four weeks of treatment.

Case 2

**Figure 3:**
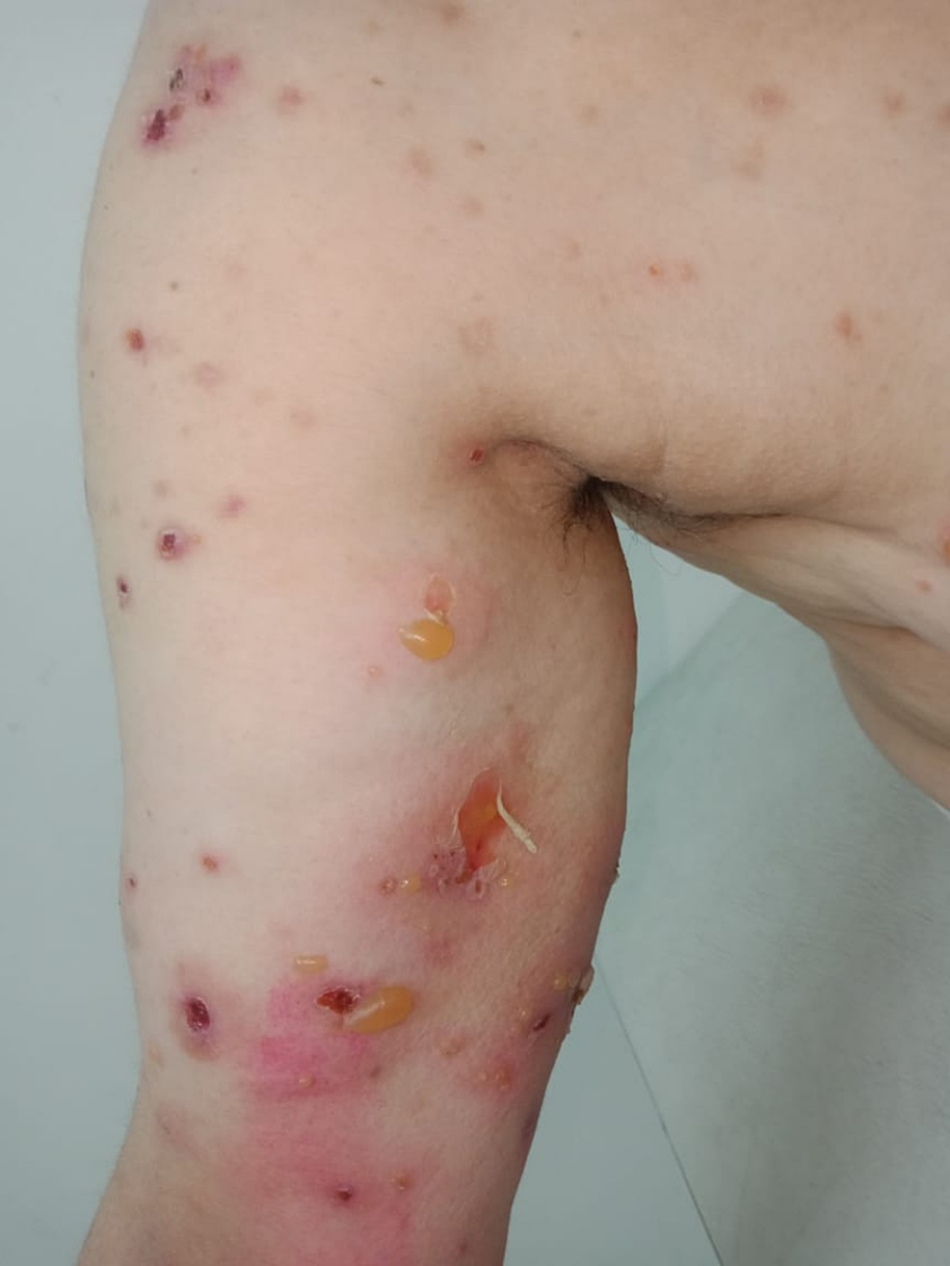
Tense bullae associated with erosions on the left arm with a positive Nikolsky sign

A 54-year-old woman presented with a one-week history of a widespread erythematous rash and blistering at the site of vaccination. She had received the first dose of the COVID-19 vaccine (Oxford AstraZeneca) administered into the left upper arm three days before the onset of the rash. She had been previously well with no specific medical history. The rash had begun at the injection site with small localized blisters. It had then spread from the left upper arm to the abdomen, back, and lower legs. Physical examination on admission revealed tense bullae associated with urticarial plaques on both arms, abdomen, and thighs, with a positive Nikolsky sign (Figure 3).

She also had erosions on the mouth palate. Histological examination showed subepidermal split with a superficial perivascular inflammatory infiltrate and predominant eosinophils. Both direct and indirect immunofluorescence were positive with linear IgG and C3, consistent with BP. Biological examinations revealed an eosinophil count of 2200/mm^3^. According to the previously cited pharmacovigilance protocol [3], the causality score assessment was I5B4 for the vaccine, highly suggestive of a diagnosis of vaccine-induced BP. The patient was put on topical corticosteroids with good evolution (clobetasol propionate 0.05%). The vaccine booster was not contraindicated but was refused by the patient.

Case 3

**Figure 4:**
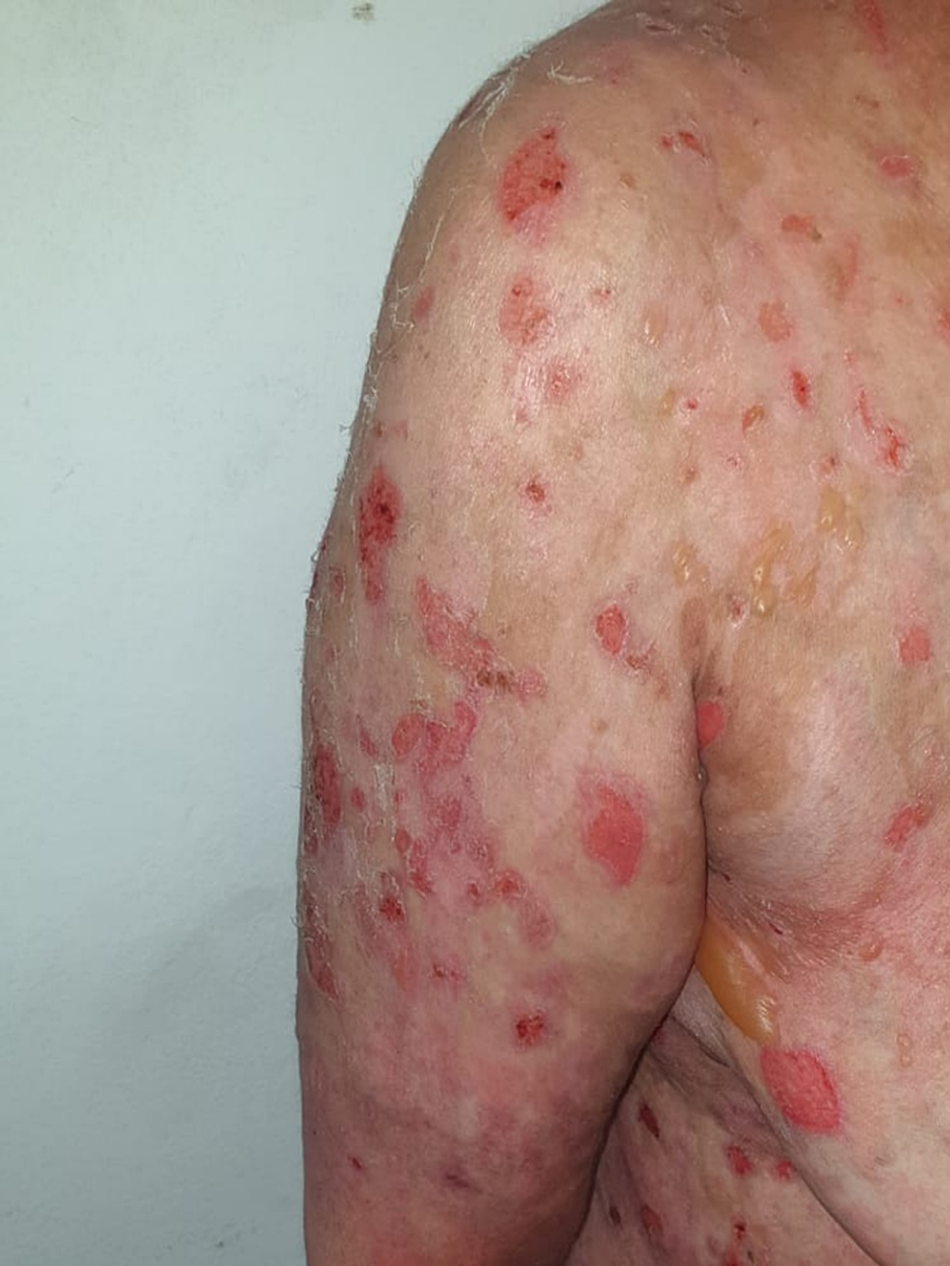
Several erosions on the erythematous base on the trunk with a positive Nikolsky sign

The patient was a 68-year-old man with a history of hypercholesterolemia for 20 years with no other particular personal or familial history. Two weeks after receiving the first dose of the Oxford AstraZeneca COVID-19 vaccine, he developed blisters at the vaccination site, which was initially misdiagnosed as post-traumatic lesions. Seven days after he received the vaccine booster, which had been administered within three weeks of the first dose, the symptoms were exacerbated with the appearance of new blisters on his limbs and trunk. Upon physical exam, we found tense bullae and post-bullous erosions on an erythematous base measuring 3-10 cm in diameter on trunk and limbs, with a positive Nikolsky sign (Figure 4).

Erosions on the oral and genital mucosa were also observed. The rest of the clinical examination was normal. Blood exams were carried out, which returned normal except for high eosinophilia at 2750/mm^3^. The histological examination showed a subepidermal blister with an inflammatory infiltrate of eosinophils. Direct immunofluorescence revealed a linear C3 pattern along the basement membrane; a diagnosis of BP was made. The imputability score was I5B4, highly suggestive of vaccine-induced BP. The patient required oral corticosteroid therapy at 30 mg/day (0.5 mg/kg/day) for a month, which led to a favorable outcome; a progressive degression was then started.

Case 4

**Figure 5:**
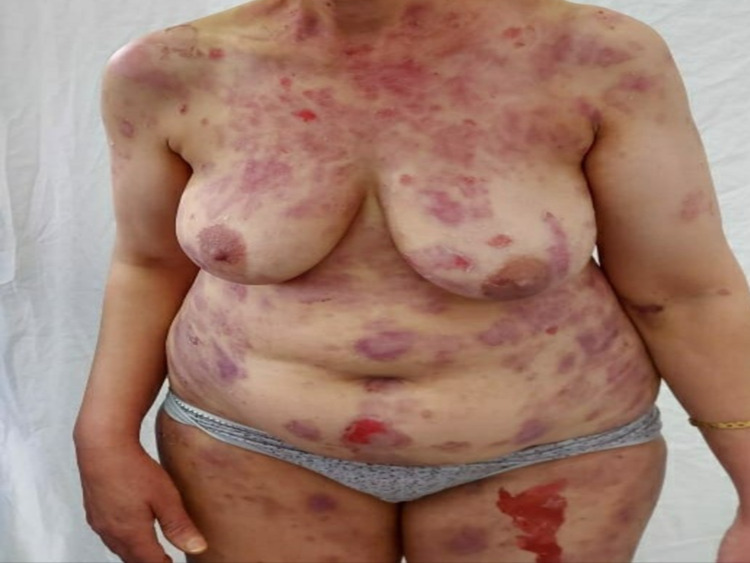
Post-bullous erosions, especially on the trunk, with a positive Nikolsky sign

A 50-year-old woman was admitted for the management of full-body erosions that had appeared 15 days after the administration of the Pfizer/BioNTech COVID-19 vaccine. She had no specific medical history. Clinical examination revealed extensive post-bullous erosions, especially on the trunk, back, and scalp (covering 75% of the skin area), with a positive Nikolsky sign (Figure 5).

She had no mucosal involvement. Histological examination showed a superficial epidermal blistering process with intact basal layer and intraepidermal eosinophils. Direct immunofluorescence showed intracellular IgG and C3, and indirect immunofluorescence was positive for anti-intercellular substance antibodies, indicating foliaceous pemphigus. The pharmacovigilance investigations determined a causality score of I5B4 for the vaccine, highly suggestive of a diagnosis of vaccine-induced pemphigus. The patient was put on oral corticosteroid therapy at 65 mg/day (1 mg/kg/day), with a complete resolution of lesions after only three weeks of treatment.

Case 5

**Figure 6:**
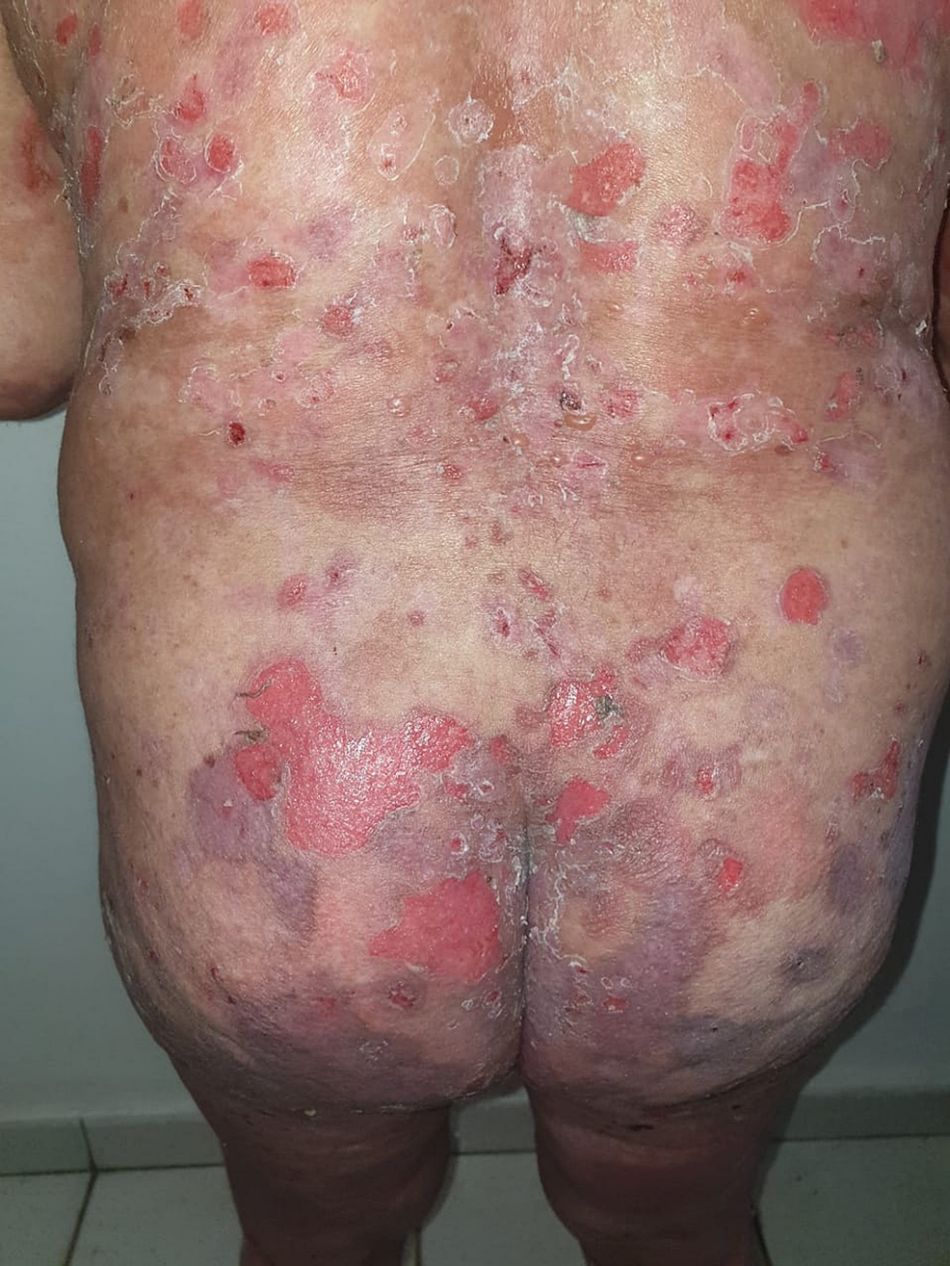
Post-bullous erosions, especially on the trunk, with a positive Nikolsky sign

A 58-year-old woman with a 10-year history of depression treated by sertraline presented one month after the administration of the first dose of the Pfizer/BioNTech COVID-19 vaccine with erosions all over her body, necessitating hospitalization. Physical examination revealed extensive erosive lesions on her face, trunk, abdomen, back, buttocks, and both legs (Figure 6).

She also had erosions on the oral and genital mucosa. A histological examination of an abdominal lesion revealed intraepidermal vesicle with acantholysis, and diffuse perivascular dermal lymphocytic and eosinophilic infiltration. Direct immunofluorescence demonstrated deposit of IgG and C3 on epidermal cell surface membranes. Based on these findings, a diagnosis of pemphigus Vulgaris was made. The imputability of both the vaccine and sertraline was studied and the investigations implicated the vaccine. The patient's condition improved with corticosteroid therapy at 55 mg/day (1 mg/kg/day). She is still scheduled to have her booster shot.

Table 1 summarizes the data of the described patients with new-onset AIBD following COVID-19 vaccination.

**Table 1:** Patients' characteristics COVID-19: coronavirus disease 2019; AIBD: autoimmune bullous disease

Patients	Age in years/sex	Implicated vaccine	Onset time	AIBD	Mucosal involvement	Skin surface affected	Eosinophils count	Treatment
Patient 1	51/M	Second dose of Oxford AstraZeneca COVID-19 vaccine	7 days	Bullous pemphigoid	Yes	70%	3900/mm^3^	Corticosteroid therapy 0.5 mg/kg/day
Patient 2	54/F	First dose of Oxford AstraZeneca COVID-19 vaccine	3 days	Bullous pemphigoid	Yes	30%	2200/mm^3^	Dermocorticoids
Patient 3	68/M	Second dose of Oxford AstraZeneca COVID-19 vaccine	7 days	Bullous pemphigoid	Yes	50%	2750/mm^3^	Corticosteroid therapy 0.5 mg/kg/day
Patient 4	50/F	Second dose of Pfizer/BioNTech COVID-19 vaccine	15 days	Foliaceus pemphigus	No	75%	500/mm^3^	Corticosteroid therapy 1 mg/kg/day
Patient 5	58/F	First dose of Pfizer/BioNTech COVID-19 vaccine	1 month	Pemphigus Vulgaris	Yes	80%	850/mm^3^	Corticosteroid therapy 1 mg/kg/day

## Discussion

The onset of AIBD may be generated by a variety of factors, mainly by creating a hyper-stimulated state of the immune system [1]. Vaccines form a substantial component of the environmental factors that affect the immune system. The combination of a genetically predisposed status in an individual with a hyper-stimulated state of the immune system may trigger an AIBD. Cross-reactivity due to the resemblance between immunogenic proteins on the severe acute respiratory syndrome coronavirus 2 (SARS-CoV-2) virus membrane and human extracellular molecules might be a plausible trigger [4].

BP is characterized by the appearance of tense blisters on an erythematous base and the existence of circulating IgG against BP180 and BP230 antigens of hemidesmosomes [5]. However, the role of vaccines in the onset of BP is not completely elucidated. The main mechanism by which vaccines, especially inactivated vaccines (such as the Oxford AstraZeneca COVID-19 vaccine in our cases), provide immunity involves the humoral pathway by the stimulation of B-lymphocytes, leading to the production of antibodies against the structural proteins [6]. Moreover, the inflammation caused by vaccination may lead to a disruption of the basement membrane, followed by the subsequent production of anti-basement membrane-specific antibodies [7]. Only a few cases of BP following vaccination have been reported in the literature in adults and children [8]. Based on our observations and a review of the literature [9], the induced BP is characterized by large bullae, a positive Nikolsky sign, mucosal involvement, and high eosinophil count, which were all found in our patients.

In the case of pemphigus, the circulating autoantibodies bind to transmembrane proteins involved in desmosome assembly (desmogleins), leading to acantholysis [10]. In addition to the mechanism of molecular mimicry, mRNA vaccines (such as the Pfizer/BioNTech COVID-19 vaccine, which had been administered to our patients with pemphigus) may give rise to a cascade of immunologic events, ultimately leading to aberrant activation of the innate and acquired immune system [11]. This may be particularly true in young women, due to the overexpression of X-linked genes that mediate the antiviral response and the stimulatory effect of estrogen on the immune system. Our pemphigus patients were all female [12].

Kasperkiewicz et al. investigated the link between vaccines against SARS-CoV-2 and the evolution of autoimmunity by testing serum samples for autoantibodies to the main immunobullous autoantigens (desmoglein 1, desmoglein 3, envoplakin, BP180, BP230, and type VII collagen) on 12 seropositive post-COVID-19 individuals and 12 seropositive healthy volunteers who received two doses of the Pfizer-BioNTech mRNA COVID-19 vaccine. Although the results argued against a relationship between SARS-CoV-2 infection/vaccines and AIBDs, the theory of disease-triggering antibody cross-reactivity was highly suggested [13].

## Conclusions

These vaccine-induced manifestations can probably be attributed to either cross-reactivity between autoantigens and the antigens injected with the vaccine, or simply to the effects of the adjuvants. We hypothesize that vaccination against COVID-19 triggers an immunological response in genetically predisposed individuals. This type of dysregulation is then reinforced by other autoimmune mechanisms. COVID-19 vaccine probably needs to be added to the list of vaccine triggers of AIBD.

However, given the complications of SARS-CoV-2 infection, the uncertainty of the causality between vaccinations and adverse events, and the rarity of these events, clinicians should still encourage full vaccination against COVID-19, including the completion of boosters in those with induced blisters after the first and second doses. A very interesting aspect we observed is that patients with new-onset BP had been administered inactivated COVID-19 vaccines while the patients with induced pemphigus were diagnosed following mRNA COVID-19 vaccinations. This might be a simple coincidence given that a case of BP following the mRNA COVID-19 vaccination has been recently reported.
